# Client satisfaction with existing labor and delivery care and associated factors among mothers who gave birth in university of Gondar teaching hospital; Northwest Ethiopia: Institution based cross-sectional study

**DOI:** 10.1371/journal.pone.0210693

**Published:** 2019-02-06

**Authors:** Kiros Terefe Gashaye, Adino Tesfahun Tsegaye, Getachew Shiferaw, Abebaw Gebeyehu Worku, Solomon Mekonnen Abebe

**Affiliations:** 1 Department of Obstetrics and Gynecology, School of Medicine, College of Medicine and Health Sciences, University of Gondar, Gondar, Ethiopia; 2 Department of Epidemiology and Biostatistics, Institute of Public Health, College of Medicine and Health Sciences, University of Gondar, Gondar, Ethiopia; 3 Department of Reproductive Health, Institute of Public Health, College of Medicine and Health Sciences, University of Gondar, Gondar, Ethiopia; 4 Department of Nutrition, Institute of Public Health, College of Medicine and Health Sciences, University of Gondar, Gondar, Ethiopia; Monash University, AUSTRALIA

## Abstract

**Background:**

There are many reasons for mothers not receiving modern obstetric care, being dissatisfied by health care deliveries is one of the major factors. There are limited studies about maternal satisfaction with labor and delivery care services in Ethiopia and particularly in the study area. Therefore, the aim of this study was to better understand client satisfaction on existing labor and delivery care service and associated factors among mothers who gave birth in the University of Gondar Teaching Hospital, Ethiopia.

**Methods:**

This institution based cross-sectional study was conducted at the University of Gondar Referral Hospital. 593 mothers who gave birth between July and September 2016 were enrolled. Study participants were selected by systematic random sampling. A standardized, interviewer-administered questionnaire was used to collect data. Descriptive and summary statistics were performed. A linear regression model was fitted and variables having a P value of ≤0.05 in the multivariable model were considered statistically significant.

**Result:**

Overall, 31.3% of mothers were satisfied by the existing labor and delivery care. Living in rural areas **(-2.9%; 95% CI: -5.75,-0.12)** and the presence of a co-morbidity **(-3.2%; 95%CI:-5.70, -0.72)** were the factor which have a negative influence on maternal satisfaction. On the other hand, travel time to reach to the hospital (hours) **(0.79%; 95% CI: 0.07, 1.52)**, birth by episiotomy or assisted vaginal delivery **(6.3%; 95%CI: 1.56, 11.04)**, and receiving cost-free maternal health services **(6.66**%**; 95%CI: 3.31, 10.01)** were the factors that had positive influence.

**Conclusion:**

The level of satisfaction of laboring mothers with the labor and delivery care services was poor. Rural residency and chronic medical co-morbidity were negatively associated with level of satisfaction while travel time, mode of delivery, and payment free delivery service had a statistically significant positive influence on satisfaction.

## Background

Client satisfaction is defined as the individual’s positive evaluation of distinct dimensions of health care, particularly when patient expectations are met [1,2]. Patients’ perception of service quality shapes their confidence with regard to the use of the available healthcare facility. Patients with lower expectations tend to be more satisfied. Efficient, quality care that meets patients’ expectations is a fundamental aim of public health services.

Women’s satisfaction with maternal health care services is integral to the current push to decrease maternal mortality; especially in the developing world, particularly Sub Saharan Africa including Ethiopia. Currently, many countries are undergoing an obstetric transition, gradually shifting from a pattern of high maternal mortality and high fertility to low maternal mortality and low fertility through utilization of modern obstetric care and family planning services[3].

According to the Mini EDHS of 2014, Ethiopia is one of the countries with very high fertility rate (4.1), with rural women having twice as many children as urban residents. Maternal health coverage is very poor with at least one visit ANC follow up of 41%, skilled birth attendance of 16% and postpartum care of 13% [4]. According to the WHO, Ethiopia is one of the developing countries with very high maternal mortality with a ratio of 420 per 100,000 live births[5].

Shifting traditional methods of labor and delivery management at home, towards the modern medical care of maternal health issues within institutions is the preferred method of reducing both maternal and perinatal morbidity and mortality [3,6–9].

Though modern obstetric care has been shown to be effective in reducing maternal mortality and physical harm, the public health system has been accused of abuses of patients’ rights. They have been shown to be deficient in treating the psychological component of clients, with these shortfalls often manifesting as poor patient satisfaction, especially in resource-limited countries [9–11]. Studies show that women accessing modern institutional health care still face many challenges including disrespectful, abusive, and inhumane ways of treatment, especially during labor and delivery processes. Such treatment violates the right of women to respectful care, and can also threaten their rights to life, health, bodily integrity, and freedom from discrimination [9,11–15].

Evidence has shown that dissatisfied mothers, especially in the developing world like Ethiopia, tend to prefer utilizing traditional means of health care, using modern health care services as a last resort [14,16].

For this reason, reproductive health experts advocate that health institutions delivering maternal health care services should strive towards client interest so that pregnant mothers will develop trust and confidence in the utilization of the health system, with the goal of reducing maternal mortality.

Although the determination of satisfaction is a complex and dynamic process [15,17,18], available evidence shows that the degree of client satisfaction and its determinants in the labor and delivery process are more closely measured in the developed world than in developing nations, including Ethiopia [16,19]. This study aims to estimate the degree of patient satisfaction and its determinants in labor and delivery care in the University of Gondar Hospital. These findings may help health managers and policy makers in making maternal health services more women-friendly.

## Methods

### Study design and setting

This institution based cross-sectional study was conducted at the University of Gondar Hospital in Northwest Ethiopia between July and September 2016. The University of Gondar Hospital provides referral and primary maternal health services for an estimated population of more than five million. Currently, the hospital holds 550 beds, of which 58 beds serve for obstetric admissions. It handles 8000–9000 deliveries per year. On top of this, the Hospital is a teaching Hospital where thousands of students interact with the patients round the year.

### Participants

The study population included all mothers who delivered at University of Gondar Hospital from July through September 2016. Mothers who were critically ill and unable to communicate were excluded. Sample sizes were calculated using data from a previous study conducted nearby in 2014 (Table 1).

**Table 1:** Sample size determination.

Place of study	Prevalence in previous study	Confidence Interval	Margin of error	Calculated sample size including 5% none response rate
Intrapartum	Amhara-61.9%	95%	4%	**593**
	Arbaminch-90.2%	95%	3%	396

Our final sample size was 593.

### Study variables

The dependent variable was maternal satisfaction and the independent variables were patient-related factors (age, level of education, wealth status, cost of care, co-morbidity, plan of pregnancy, ANC visit, duration of stay in the facility, duration of labor, mode of delivery, time of delivery), health professional related factors (sex of professionals, type of professionals, number of caregivers), and health facility-related factors (distance from the health facility).

### Operational definitions

Overall satisfaction level: satisfaction was measured using a 36 item questionnaire developed by the WHO and Ethiopian Federal Ministry of Health. Participants were labeled as “satisfied” when they scored75% and above, and they were labeled as “unsatisfied” when they scored less than 75% [17,19,20].

Spontaneous vaginal delivery (SVD):- Vaginal delivery with no intervention other than perineal support.

Care: In this study, care was defined as the service provided to laboring women starting from the entrance of the hospital gate until the time of discharge from labor and delivery ward.

Satisfaction by welcoming environment of the health institution: the perception of mothers about the care given by the hospital staff from entry to the hospital gate until admission to the labor ward.

### Data collection procedures

The data was collected using a standardized questionnaire with an exit interview of clients that come to the University of Gondar Hospital for delivery services. The questionnaire was designed to obtain information including socio-demographic characteristics, reproductive history, satisfaction, and factors affecting satisfaction of service users. For the measurement of satisfaction, a standardized tool developed by the World Health Organization (WHO) and the Federal Ministry of Health (FMOH) of Ethiopia with 36 items was used. The questionnaire was prepared in English and translated into Amharic by a professional translator. Four nurses who did not work in the labor and delivery ward collected the data under the supervision of public health professionals. Data collectors and supervisors received training about procedures of the study prior to data collection. The questionnaire was pretested using a small sample not included in the final analysis. Data completeness was checked daily.

### Data processing and analysis

The collected data were entered into Epi-info version 3.5.3 and then transferred into STATA version 14.0 software for analysis. The results were organized, summarized and presented using appropriate descriptive measures. A principal component analysis was also used to classify the wealth index as the lowest, middle, and highest. The outcome variable was categorized using 75% as a cut-off value. Those participants who scored more than or equal to 75% were categorized as satisfied and those scored below were leveled unsatisfied. For the purpose of identifying determinant factors, ‘satisfaction’ was considered as a continuous variable. A linear regression model was fitted to identify factors associated with client satisfaction. Reliability analysis was performed to ensure that items with in each composite were consistent. Internal consistency/reliability was checked by calculating Cronbach’s alpha for each of the composites to examine the extent to which respondents answered consistently to the theoretically similar items in each composite. Before fitting the linear regression model, the assumptions were checked. The assumption of linearity was checked and satisfied using both a scatter plot and lack of fit test. The assumption of normality was checked by plotting histogram and P-P plots. The assumption of homoscedasticity was satisfied by plotting to scatter plot of standardized residuals against the standardized predicted values and it was randomly distributed.

The Durbin Watson statistic was used to check the assumption of independence of errors and autocorrelations. The value of the Durbin Watson statistic for this data was 2.00 which fall within the acceptable range from 1.50 to 2.50; therefore, this analysis satisfied the assumption of independence and no autocorrelations. Multicollinearity assumption was checked through the Variance Inflation Factor (VIF). The analysis showed VIF for each independent variable less than 10. Hence there was no evidence of multicollinearity. Enter method was used to fit the multivariate linear regression model. Variables that had a P value of ≤0.05 in the multivariable model were considered as statistically significant.

The 36 satisfaction measuring items were categorized into three subsets to describe the major areas of weakness and strength within the hospital service. These subsets included health care provider related (welcoming gesture, listening to worries, explanation, and counseling of care, consent, respect, empathy etc.), service related (privacy, freedom of movement, presence of birth companion, pain management etc.)and hospital infrastructure related (ease of access to the maternity ward, cleanliness, availability of toilet, bathroom and electric supplies, adequacy of space, attractiveness of the ward etc.)variables.

### Ethical considerations

Ethical clearance was obtained from the College of Medicine and Health Sciences, University of Gondar, Research Ethical Review Committee. A formal permission letter was received from University of Gondar Hospital Management team (University of Gondar teaching and referral hospital clinical director office). Written consent was also obtained from each study participant after the objectives of the study were explained to them. For mothers in the age group of between16 to 18, parent and/or guardian consent was waived by the research ethics committee. Study participants were interviewed in a private place, and the data were kept confidential.

## Results

### Socio-demographic characteristics

A total of 579 participants were involved in the study with a response rate of 97.6%. The mean (±SD) age of the participants was 26.8 (±5.3) years with a range of 16 to 44 years. There were 4 study participants under the age of 18 (one 16 and three 17). The majority (88.3%) was below the age of 34 years. Eighty-eight percent of respondents described themselves as Orthodox Christians, while 11.9% were Muslims. Five hundred sixty-three of the respondents (97.2%) were married and 479 (82.7%) were urban dwellers (Table 2).

**Table 2:** Socio-demographic characteristics of laboring mothers in the University of Gondar Hospital, Northwest Ethiopia, August 2016.

Variable	Frequency	Percentage (%)
**Age in years**		
<20	28	4.84
20–34	483	83.42
35–49	68	11.74
**Residence**		
Urban	479	82.73
Rural	100	17.27
**Religion**		
Orthodox	511	87.95
Muslim	69	11.88
Protestant	1	0.17
**Ethnicity**		
Amhara	553	96.17
Tigrie	8	1.39
Kimant	13	2.26
Other	1	0.17
**Marital status**		
Married	563	97.24
Unmarried	16	2.76
**Maternal educational status**		
No Formal Education	166	28.67
Primary Education	106	18.31
Secondary Education	156	26.94
Tertiary Education	151	26.08
**Partners educational status**		
No Formal Education	157	27.12
Primary Education	87	15.03
Secondary Education	126	21.76
Tertiary Education	209	36.1
**Maternal Occupation**		
Housewife	**317**	**54.75**
Employed	**139**	**24.01**
Merchant	**76**	**13.13**
Daily laborer	**16**	**2.76**
Farmer	**15**	**2.59**
Student	**16**	**2.76**
**Wealth index**		
Lowest	206	35.58
Middle	216	37.31
Highest	157	27.12

### Reproductive history and health status of participants

The mean age of participants at the first birth was 22 years (SD±3.92). Among all the participants, 531 (91.7%) mothers’ current pregnancies were described as wanted. 542 (93.6%) of the mothers had ANC follow-up for the current pregnancy, and among them, 187 (34.5%) were followed in UoG Hospital (Table 3).

**Table 3:** Reproductive history and health status of laboring mothers in the University of Gondar Hospital, Northwest Ethiopia, 2016.

Variable	Frequency	Percentage (%)
**History of Parity**		
Primipara	276	47.67
Multipara	276	47.67
Grand multipara	27	4.66
**History of stillbirth**		
No	530	91.54
Yes	49	8.46
**Duration of birth spacing**		
9months-2years	54	17.82
2–5 Years	155	51.16
>5 years	94	31.02
**Place of delivery for the last child**		
UoG Hospital	128	42.24
Another Health facility	81	26.73
Home	94	31.02
**Wontedness of current pregnancy**		
Yes	531	91.71

Gynecology and Obstetrics residents attended the majority of deliveries, 301 (52%), followed by medical interns attending 140 (24.2%) (Table 4). Regarding the mode of delivery, 244 (42.14%) were spontaneous vaginal deliveries (SVD), followed by cesarean delivery 159 (27.5%), episiotomy assisted delivery 150 (25.9%), and instrumental 26 (4.5%). Five hundred thirty-nine (93.1%) of the mothers paid for drugs and materials used in their delivery process.

**Table 4:** Category of health professionals and number of laboring mothers they followed in University of Gondar Hospital, Northwest Ethiopia, August 2016.

Health professional followed laboring mothers	Frequency	Percentage
^#^Intern	338	58.38%
Intern with resident	94	16.23%
Midwives	55	9.50%
Midwife and intern	36	6.22%
*Resident	17	2.94%
Midwife, intern, and resident	15	2.59%
Senior obstetrician and gynecologist	14	2.42%
Intern with Student	3	0.52%
Intern, resident, and specialist	3	0.52%
**Students	2	0.35%
Midwife with resident	1	0.17%
Intern, student, and resident	1	0.17%
Total	579	100.00%

** Students = students who came to the ward for learning (other than interns and residents)

^#^ Intern = graduating class medical students

*Resident = gynecology and obstetrics residents

### The overall level of satisfaction

Out of the total participants, 181 (31.3%) of the mothers were satisfied with the labor and delivery care (had a satisfaction score of ≥75%). The welcoming environment of the institution made 551 (95.16%) of the respondents satisfied and 342 (59.07%) were also satisfied by the presence of companionship in the delivery room. Three hundred thirty-seven (58.20%) of the women were satisfied by the privacy during the pelvic examination but only 223 (38.5%) were satisfied by the freedom of movement in the ward during labor and delivery. The majority (86.87%) of the clients were also satisfied with the availability of professionals when they needed them.

**Fig 1:**
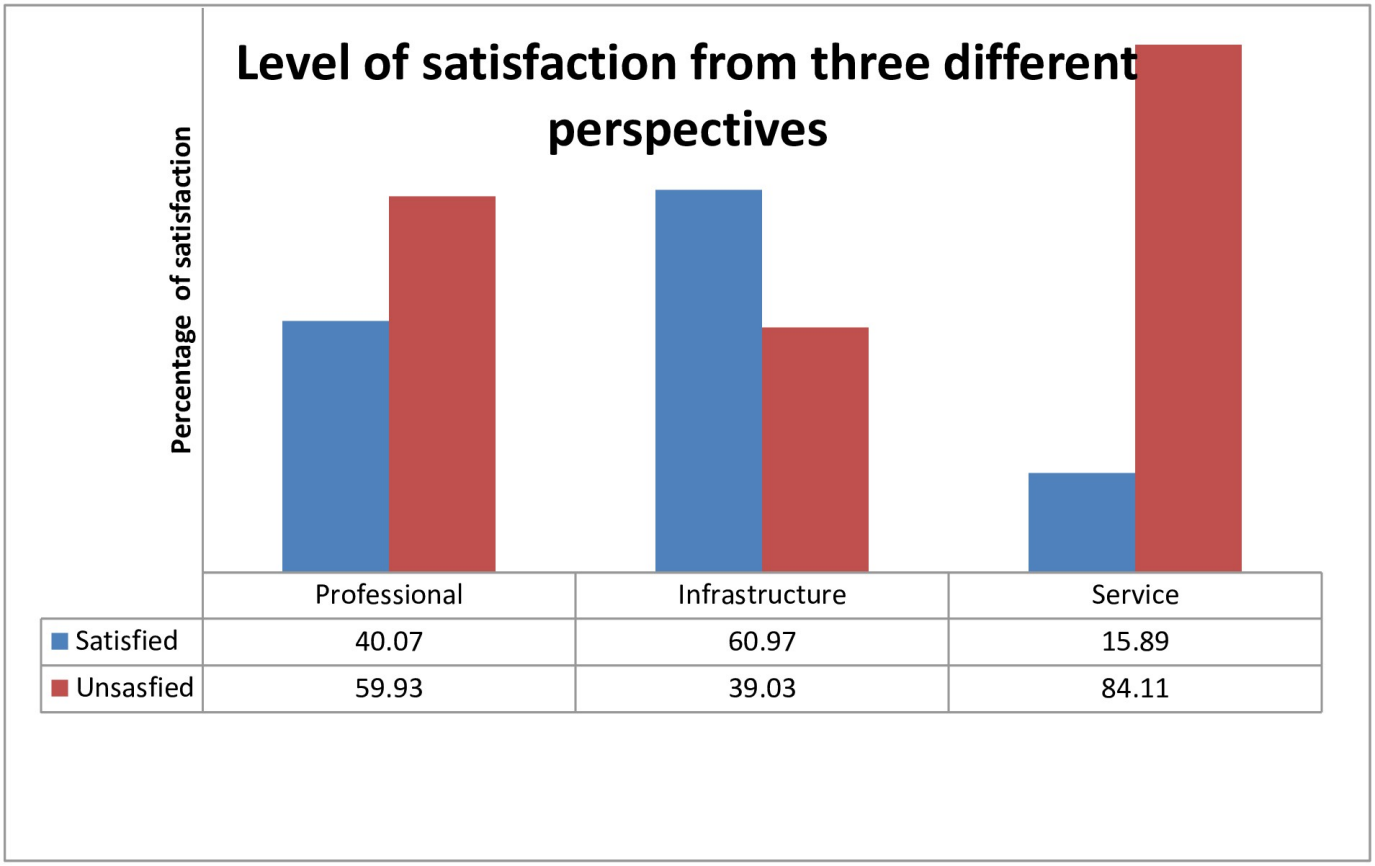
Level of satisfaction of laboring mothers by health professional behavior and communication, institutional infrastructure, and service at University of Gondar Hospital, Northwest Ethiopia, 2016.

Satisfaction in each of the three categories was as follows: 40.07% were satisfied with health professional behavior and communication, 60.97% were satisfied with institutional infrastructure, and 15.89% were satisfied with the service (Fig 1).

**Fig 2:**
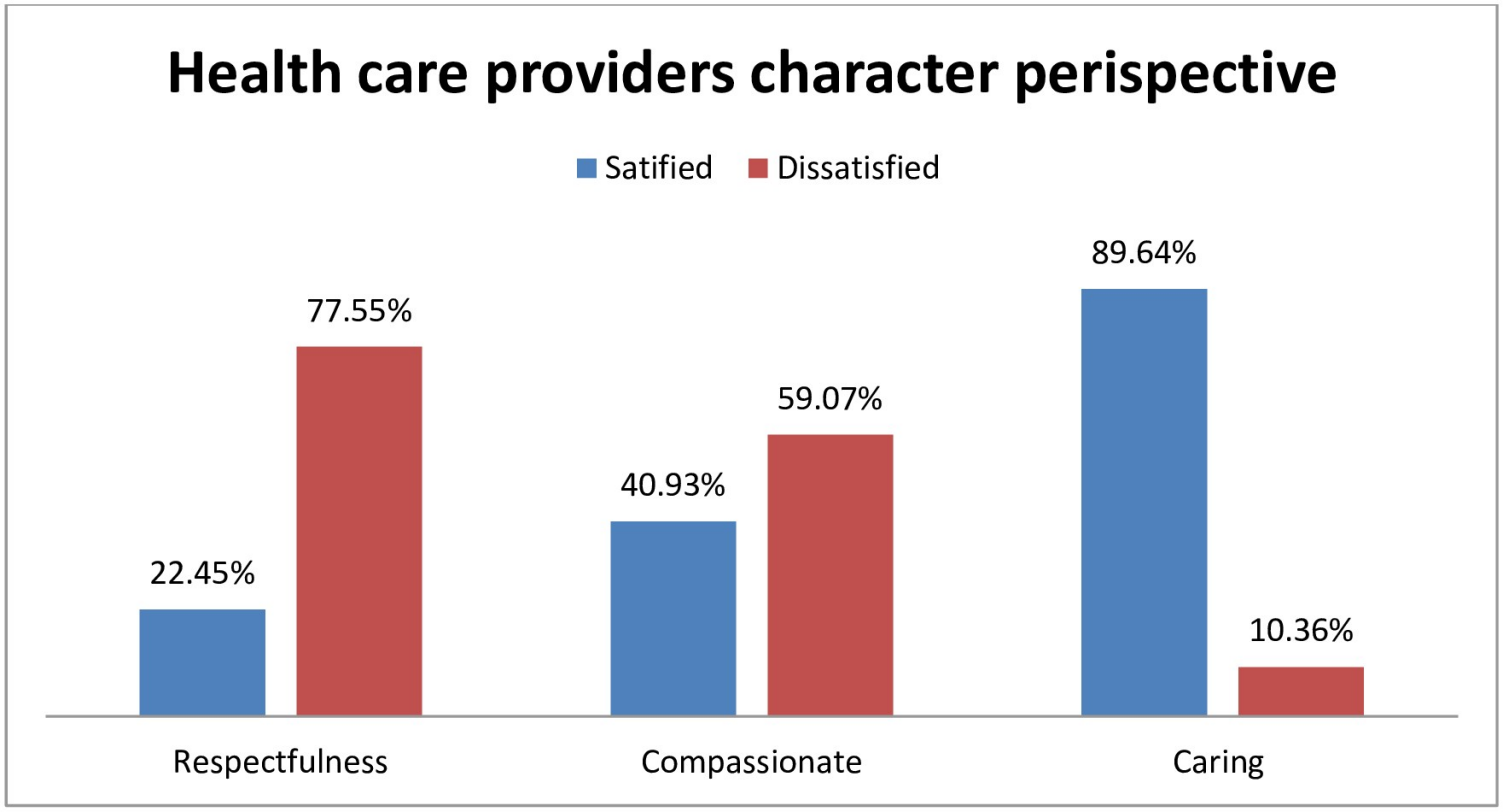
Level of satisfaction of laboring mothers by health professionals respectfulness, compassionate and caring behavior at University of Gondar Hospital, Northwest Ethiopia, 2016.

From indicators of health professional behavior and communication, only 130 (22.45%) were satisfied by professionals’ respectfulness, 237 (40.93%) by their compassion, and519 (89.64%) by their caring nature (Fig 2).

### Factors associated with maternal satisfaction

In the multivariable linear regression model, rural residency and chronic medical co-morbidity had a negative influence on the level of satisfaction while travel time, mode of delivery, and payment free delivery service had a statistically significant positive influence on satisfaction (Table 5).

**Table 5:** Multivariable linear regression indicating factors associated with satisfaction of mothers by labor and delivery care service at UoG Hospital,2016.

Variable	Crude B coefficient (95%CI)	Adjusted B coefficient (95%CI)	P- value
**Age**	0.10 (0.00, 0.20)	0.03(-0.19, 0.25)	0.785
**Place of residence**			
Urban	**0**	**0**	
Rural	**0.80 (0.60, 2.13)**	**-2.93(-5.75,-0.12)**	**0.04**
**Distance in hours**	**0.42 (0.11, 0.74)**	**0.79 (0.07, 1.52)**	**0.03**
**Marital status**			
Married	2.62 (-0.50, 5.74)	0.19 (-5.62, 5.99)	0.95
Not Married	0		
**Wealth index**			
Lowest	1.13 (-0.20, 2.43)	1.65 (-0.59, 3.89)	0.15
Middle	0.60 (-0.63,1.76)	0.69(-1.18, 2.57)	0.47
Highest	0	0	
**Wontedness of current pregnancy**			
Wanted	0.16 (-1.70, 2.01)	-0.15 (-2.99, 2.68)	0.91
Unwanted	0	0	
**History of ANC for current pregnancy**			
Yes	-1.99 (-4.07, 0.10)	-3.33 (-11.03, 4.37)	0.39
No	0		
**The total duration of labor**			
0–12 hours	-0.29(-3.64, 3.10)	4.52 (-1.55, 10.59)	0.14
12–24 hours	-1.10 (-4.51, 2.30)	5.00 (-1.17, 11.18)	0.11
24–48 hours	0.72 (-2.95, 4.40)	5.18 (-1.46, 11.81)	0.12
**Mode of delivery**			
SVD	1.39 (-1.13, 3.91)	3.56 (-0.92, 8.04)	0.12
Episiotomy Assisted	**1.96 (-0.64, 4.55)**	**6.30 (1.56, 11.04)**	**0.009**
Cesarean delivery	3.23(0.65, 5.82)	2.39 (-2.11, 6.88)	
Instrument-assisted	0	0	
**Duration of Hospital stay during labor**			
< 6 hours	1.7(0.4, 3.0)	1.26 (-1.32, 3.85)	0.34
6–12 hours	0.97 (-0.3, 2.3)	1.40 (-1.04, 3.84)	0.26
>12 hours	0	0	
**Payment for the delivery process**			
Free	**4.08 (2.09, 6.07)**	**6.66 (3.31, 10.01)**	**0.000**
Paid	**0**	0	
**Co-morbidity**			
Yes	**-1.35(-3.01, 0.32)**	**-3.21(-5.70, -0.72)**	**0.01**
No	**0**	0	

Living in a rural area was associated with a 2.9% **(-2.9; 95% CI: -5.75,-0.12)** decrease in satisfaction score compared with those who live in an urban environment. An hour unit increase in time required to reach the hospital increased the satisfaction score by 0.79% (**0.79%: 95%CI: 0.07, 1.52)**.

Episiotomy assisted vaginal delivery was associated with a 6.3% **(6.3: 95% CI, 1.56, 11.04)** increase in satisfaction score compared with those attended by instrumental vaginal delivery. Obtaining free service was associated with a 6.66% (**6.66; 95% CI: 3.31, 10.01)** increase in satisfaction score than who paid for drugs and supplies. The presence of co-morbidity was associated with a 3.2% (**-3.21; 95%CI: -5.70, -0.72)** decrease in satisfaction score compared with those who did not have a co-morbidity.

## Discussion

Institutional delivery is one of the best strategies to decrease the very high maternal mortality rates in Ethiopia and other developing countries. To achieve it, promoting quality maternal health services with a better client satisfaction is vital.

In this study, the overall satisfaction of mothers who delivered in the study area was 31.3%, which is very low when compared with other studies done in countries like the USA (83%), Australia (73%), Sweden (67%), Iran (59.5%), South Africa (91.2%), Malawi (97.3%) and Kenya (56%)[15,20–27]. This variation may be due to a real difference in the quality of services provided, study setting or socio-economic characteristics of the population. Moreover, unlike this current study setting, those countries have better infrastructure, more trained health professionals, and more educated people; these factors might play a great role in having a higher level of maternal satisfaction of childbirth health care.

The maternal satisfaction rate observed in this study is also very low when compared with other studies conducted in Ethiopia, such as researches in Debremarkos town 81.7%, Assela Hospital 80.7%, and in three referral hospitals in Amhara region, including UoG Hospital 61.9%[17,27,28]. This may be due to differences in methodology, or to a real difference in the quality of services provided. When the study in Debre Markos Referral Hospital was conducted, the hospital had received a national quality award by the Federal Ministry of Health (FMOH), and it may have affected the quality service provided. The difference from studies done in Assela, Dessie, and Felegehiwot hospitals might be due to the difference in the hospitals’ settings. Unlike the other studies, this current study was conducted in a large teaching hospital where a large number of trainees/students learn and/or practice on clients. These factors may contribute to the lower level of satisfaction in the hospital.

However, maternal satisfaction in this study is higher than the rate observed in Jordan, 17.8% [15]. This variation may be due to a real difference or a difference in methods, study setting and population. It is also better than previous studies done in Ethiopia including a study done in Addis Ababa, Gandhi Memorial Hospital with 21% [29] which may be due to differences in study setting where Gandhi Memorial Hospital is the busiest hospital in delivering maternity care in the country. Residents of Addis are more educated and more knowledgeable about health-related issues when compared with Gondar population. In addition, it is better than a study done in Gondar university hospital in 2011, (25.3%)[17]. This improvement may be as a result of some improvements in the infrastructure of the hospital (new maternity ward) which took the major share of satisfaction by respondents (60.97%) as well as increased number of highly qualified professionals (opening of residency program).

In an examination of important satisfaction perspectives of the labor and delivery care, the majority of the clients (95%) were satisfied by the welcoming environment of the hospital. It is a very important aspect of the labor and delivery care which ultimately encourage laboring mothers to come to the hospital for delivery service. The quality of a patient-provider relationship is very important for a positive experience of mothers during the labor and delivery care as described by a systematic review done in Canada and qualitative study done in the USA [30,31]. In this study, about 86.87% of the clients were satisfied by having professionals by their side during the labor and delivery process. This finding is in contrast to a study done in five east and south African countries where abandonment and neglect were the most frequently observed problems of professionals’ behavior [32]. When clients feel professionals are attentive to them, they will be more motivated to attend the hospital for their delivery care.

However, in this study, the majority of the clients were not satisfied with the professional’s care. This is in line with the study done in Addis Ababa where only 41.6% of participant cases reported that professionals were respectful [29], contrary to a study done in Debremarkos Town [27]. Lack of respectful care can negatively affect the hospital to be chosen as intrapartum care sites by clients, which will, in turn, increase the risk of home delivery and its potential consequences.

Privacy during the labor and delivery care, especially during pelvic examination and delivery, are important components of quality of care affecting women’s satisfaction. Lack of privacy is one of the possible reasons for laboring mothers not attending health facility. In this study, many clients were not satisfied with their privacy (41.8%), which is in line with a study done in Uganda, where the issue of privacy was one of the poor quality indicators of labor and delivery care. Additionally, three referral hospitals in Amhara region, Ethiopia reported about 53.3% of the participants were not satisfied with the privacy of the care[17,33]. However, in Debre Markos town, the assurance of privacy reached up to 98% [27].

Studies have shown that laboring mothers accompanied by family members have a positive experience with their delivery care[34–36]. In this study, many cases (59.07%) were not happy about the institutional regulation of not having a birth companion. This is in line with a study done in Japan where institutional regulations of not having a birth companion negatively affected maternal satisfaction. In addition, a study done in South Central Ethiopia reported that not having birth companion was one of the reasons for not delivering in a health facility [37,38]. The labor and delivery process is a very stressful condition in which most mothers prefer to have someone they know to share their concerns and encourage them to have a successful outcome. In addition, they can get physical and emotional support, and gaps in service provision could be full-filled by their companion. Therefore, the absence of birth companion will continue to negatively affect maternal satisfaction, and leading to the likelihood of home delivery unless improved.

It is recommended that laboring mothers assume any position except supine to maximize their comfort during labor and delivery care[8]. This is because maternal reactions including free movement are some of the mechanisms to alleviate labor pain and increase the satisfaction of mothers if they are allowed to do so. But in this study, many cases (61.5%) were not satisfied by the freedom of movement in the ward during their labor and delivery process. This is in line with the study done in Addis Ababa [29].

A study done in Ghana showed that many women prefer adequate space for movement and availability of showers, toilets, water, and electric supplies to have a better birth experience [39]. In this study, clients were shown to be relatively more satisfied with institutional infrastructure (60.97%) than both service-related conditions (15.89%) and health professionals’ behavior and communication-related conditions (40.07%). This is an improvement from a qualitative study done in the same hospital in 2009 where the issue of space was one of the key findings affecting maternal satisfaction [25,40]. This relative improvement in infrastructure may be due to the newly renovated maternity ward.

Respectful, compassionate and caring behaviors are expected from providers for increasing maternal health service satisfaction, which may lead to increasing institutional delivery and decreasing maternal mortality. Women have the right to dignified and respectful maternity care. When provider related conditions are further divided with respect to respectful, compassionate and caring approach, 22.45% of clients responded that they received respectful care which is very low when compared with a study done in Addis Ababa 88.6 to 91% [29]. However, it is higher than another study done in Addis Ababa, in Malawi, a global systematic review, Jordan, and Kenya [13,23,25,41,42]. This may be due to the very busy working environment of the maternity wards of the other studies leading providers to be more focused in avoiding life-threatening conditions, hence becoming less concerned with regard to caring and which affects women’s psychology.

The government of Ethiopia has officially declared that labor and delivery services are to be delivered for free with the goal of increasing skilled birth attendance and decreasing maternal mortality. However, in this study, the majority (93.1%) of the mothers paid for drugs and materials used for their delivery service. Clients who received free services showed a 7% increase in satisfaction score as compared to those who paid for materials and drugs. This is in line with the philosophy that free service increases institutional delivery. This can negatively affect maternal satisfaction as well as their institutional delivery as it was evidenced in studies done in South Africa and Ethiopia [17,26,38,43].

Patients from rural communities had 2.9% lower levels of satisfaction compared with urban residents. This contrasts with a study done in Addis Ababa [29] in which clients who came from rural areas were more satisfied than urban dwellers. In this study, more births from clients from rural communities were delivered through CS than among urban dweller. This may be due to the fact that most rural patients live in relatively different socio-cultural circumstances. In addition, most of them possess a low level of education. On top of this, they are new to the hospital environment. These conditions can lead to poor communication and understanding with professionals within the birth environment hence leading to poor satisfaction.

An hour increase in travel time to reach the hospital increases the satisfaction by 0.79%. This is in contrast to a cross-sectional study done in Ghana where clients who came from far away to the health facilities were less satisfied with the health care they received [44]. This may be because mothers who travel long distances to the hospital while suffering labor pain and complications will be satisfied when they get pain relief.

In this study, delivering vaginally by an episiotomy assisted procedure increases satisfaction by 6.3% as compared delivering by instruments (Vacuum and forceps). This is in contrast to a study done in Jordan where episiotomy assisted delivery was a poor predictor of satisfaction [25]. But it is in line with a study done in Brazil [35,45]. This may be due to the fact that, having a spontaneous onset of labor resulting in vaginal delivery is the natural way of human reproduction. In contrast, instrumental deliveries are more traumatic and painful ending into significant perinatal complications when compared with episiotomy assisted deliveries.

Having chronic medical co-morbidities like HIV, HTN, DM etc. is associated with a 3.1% decrease in satisfaction as compared to those who had no co-morbidity. This is consistent with a study done in Canada where having high-risk pregnancy was a negative predictor of satisfaction [46]. This may be because patients having co-morbidities might need special care and the underlying disease condition with labor pain may affect their level of satisfaction.

The information elicited from patients may not reflect the real quality of labor and delivery care. Involvement of only one health facility may not represent the satisfaction level of laboring mothers in the area. Moreover, mothers’ expectation of services provided by big hospitals like the University of Gondar hospital might potentially affect their satisfaction. Other studies utilizing observation and qualitative techniques are recommended.

## Conclusion

The overall satisfaction of laboring mothers with labor and delivery care was low. Residency, distance to the hospital, episiotomy assisted vaginal delivery, the presence of medical co-morbidity, and payment for service expense were independent determinants of the level of satisfaction. Efforts to strengthen services and to give special emphasis to mothers with chronic medical co-morbidity may be called for.
